# A New Live Auxotrophic Vaccine Induces Cross-Protection against *Klebsiella pneumoniae* Infections in Mice

**DOI:** 10.3390/vaccines10060953

**Published:** 2022-06-16

**Authors:** Miriam Moscoso, Juan A. Vallejo, Maria P. Cabral, Patricia García, Víctor Fuentes-Valverde, Eva Gato, Jorge Arca-Suárez, Pablo Aja-Macaya, Germán Bou

**Affiliations:** 1 Department of Microbiology, University Hospital A Coruña (CHUAC)—Biomedical Research Institute A Coruña (INIBIC), 15006 A Coruña, Spain; mirian.moscoso.naya@sergas.es (M.M.); juan.andres.vallejo.vidal@sergas.es (J.A.V.); m.povoa@czvaccines.com (M.P.C.); patricia.garcia.fernandez@sergas.es (P.G.); victor.fuentes.valverde@sergas.es (V.F.-V.); eva.gato@vaxdyn.com (E.G.); jorge.arca.suarez@sergas.es (J.A.-S.); pablo.aja.macaya@sergas.es (P.A.-M.); 2 Technical Services Department, CZ Vaccines S.A.U., 36400 O Porriño, Pontevedra, Spain; 3 Centro de Investigación Biomédica en Red de Enfermedades Infecciosas (CIBERINFEC), Instituto de Salud Carlos III, 28029 Madrid, Spain; 4 Vaxdyn S.L., 41704 Sevilla, Spain; 5 Intrahospital Infections Laboratory, National Centre for Microbiology, Instituto de Salud Carlos III, 28220 Madrid, Spain

**Keywords:** *Klebsiella pneumoniae*, live vaccines, D-glutamate auxotrophy, glutamate racemase, cross-protection

## Abstract

The development of a whole-cell vaccine from bacteria auxotrophic for D-amino acids present in the bacterial cell wall is considered a promising strategy for providing protection against bacterial infections. Here, we constructed a prototype vaccine, consisting of a glutamate racemase-deficient mutant, for preventing *Klebsiella pneumoniae* infections. The deletion mutant lacks the *murI* gene and requires exogenous addition of D-glutamate for growth. The results showed that the *K. pneumoniae* Δ*murI* strain is attenuated and includes a favourable combination of antigens for inducing a robust immune response and conferring an adequate level of cross-protection against systemic infections caused by *K. pneumoniae* strains, including some hypervirulent serotypes with elevated production of capsule polysaccharide as well as multiresistant *K. pneumoniae* strains. The auxotroph also induced specific production of IL-17A and IFN-γ. The rapid elimination of the strain from the blood of mice without causing disease suggests a high level of safety for administration as a vaccine.

## 1. Introduction

*Klebsiella pneumoniae*, a Gram-negative bacterium with a high genetic diversity, is widely distributed in multiple environmental and host-associated niches. This microorganism acts as an opportunistic pathogen that can asymptomatically colonize the human gastrointestinal tract and the nasopharynx. The colonization of the gastrointestinal tract by opportunistic *K. pneumoniae* strains could be considered the first step in the nosocomial infection development, from which bacteria may disseminate to other tissues, causing life-threatening infections [1]. *K. pneumoniae* strains are generally surrounded by a thick hydrophilic polysaccharide capsule (CPS) and at least 77 antigenically distinct CPSs have been recognized (K-antigens). Moreover, nine O-antigen types that are distinguished in the lipopolysaccharide of *K. pneumoniae* protect this bacterium from complement-mediated killing. Both K- and O-antigens are important virulence factors used to differentiate *K. pneumoniae* isolates. *K. pneumoniae*, which is included in the ESKAPE pathogens group responsible for the majority of nosocomial infections worldwide, has become resistant to most antimicrobial agents [2]. Additionally, according to the WHO, extended-spectrum β-lactam (ESBL)-producing and carbapenem-resistant *K. pneumoniae* are of major concern in global public health [3].

*K. pneumoniae* has evolved into two pathotypes: classical and hypervirulent (hvKp) [4]. The classical *K. pneumoniae* is the leading cause of opportunistic healthcare-associated infections, such as urinary tract infections, pneumonia, wound and surgical site infections, and bacteraemia [5]. However, severe community-acquired infections, including pyogenic liver abscess, endophthalmitis, meningitis and community-onset pneumonia, are associated with hvKp strains considered to be strict pathogens [6]. Populations at greatest risk of *K. pneumoniae* infection are neonates and elderly and immunocompromised individuals, including those with diabetes, chronic lung conditions, HIV-positive individuals and hospitalized patients. Most hvKp clones associated with invasive disease express a hypermucoid CPS type K1 or K2 and produce increased levels of siderophores [7]. Although, to date, these hvKp clones have predominantly infected individuals in Southeast Asia and South Africa [5], these infections are now increasing worldwide [6].

Some multidrug-resistant (MDR) *K. pneumoniae* clones cause localized infections within a single hospital (e.g., sequence type (ST) 70 or ST323). However, a subset of these MDR clones are widely distributed and causing outbreaks in hospital settings, especially in long-term care and pediatric units, and they have become global problematic clones. These include clonal groups (CGs) 258, CG15, ST17, CG29, CG37, ST101, CG147 and CG307. Furthermore, hvKp infections are associated with other clones, such as CG23, CG65 and CG86 [5].

Most of the described mechanisms of antibiotic resistance in *K. pneumoniae* are associated with the acquisition of large conjugative plasmids: ESBLs that provide resistance to third-generation cephalosporins and monobactams and to various serine carbapenemases (e.g., KPC, OXA-48) and metallo-β-lactamases (e.g., NMD-1, VIM, IMP) that confer resistance on almost all available β-lactams, including the carbapenem family [1,8,9,10]. In contrast to the classical *K. pneumoniae* strains, the hvKp variants are usually sensitive to most antimicrobial agents, but convergence of MDR and hypervirulence has nevertheless also been reported [5,11]. The global increase in MDR, particularly carbapenem-resistant *K. pneumoniae*, has led to the reuse of polymyxin/colistin to treat these infections (despite the reported neurotoxic and nephrotoxic side effects) or to the use of combinations with β-lactamase inhibitors, such as ceftazidime/avibactam [12]. However, the emergence and spread of plasmid-mediated resistance to colistin [13] and plasmid mutations during treatment with β-lactamase inhibitor combinations [14] have severely limited the available antimicrobial therapy options in the few last years, leading to renewed interest in vaccines against *K. pneumoniae* infections.

Currently, there is no approved vaccine for preventing *K. pneumoniae* infections, although different vaccine strategies have been explored: Uromune^®^, a whole-cell inactivated polybacterial sublingual vaccine [15]; a live attenuated vaccine based on *tonB* gene deletion, encoding an iron-uptake protein [16]; Klebvax^®^, a 24-valent *Klebsiella* CPS vaccine, which was not developed further, partly due to the wide range of clinically relevant CPS types [17,18]; conjugated vaccines linking polysaccharides to different carrier peptides (BSA, KLH, CRM197) [19,20]; and bioconjugate vaccines targeting the capsule of hvKp [21]. Vaccines based on the four most prevalent *Klebsiella* O serotypes related to human infections (O1, O2, O3 and O5) have recently been developed as conjugate and multiantigen-presenting system vaccines eliciting good immunogenicity in mice. Additional vaccine antigens (e.g., outer membrane proteins, type 3 fimbriae) and novel vaccine platforms (e.g., nanoparticles, liposomes) were also considered, as well as the addition of adjuvants [22,23].

In this study, we designed and developed a prototype vaccine consisting of a deletion mutant of *K. pneumoniae* MGH 78578 that results in D-glutamate auxotrophy. This strain shows promising potential as a live vaccine for the prevention of *Klebsiella*-induced sepsis.

## 2. Materials and Methods

### 2.1. Bacterial Strains, Growth Conditions and Plasmids

The bacterial strains and plasmids used in this study are listed in Table 1. All strains were grown in Luria–Bertani (LB) broth (10 g/L tryptone, 5 g/L yeast extract and 10 g/L NaCl) or low-salt LB (reducing NaCl to 5 g/L when hygromycin was needed) with aeration at 37 °C. For plasmid selection in *Escherichia coli*, apramycin (Duchefa Biochemie), ampicillin and hygromycin (Sigma-Aldrich, Merck Life Science S.L.U., Madrid, Spain) were added at concentrations of 50, 50 and 100 µg/mL, respectively. Apramycin and hygromycin were added to the medium at 200 µg/mL and 1250 µg/mL, respectively, to select the recombinants in *K. pneumoniae*. MGH 78578 Δ*murI* was cultivated in LB medium supplemented with 10 mM D-glutamate (Sigma-Aldrich).

### 2.2. Construction of the murI Deletion Mutant of K. pneumoniae MGH 78578

The procedure used to construct the Δ*murI* mutant strain has been reported previously [28]. The strategy was based on the use of the *E. coli* λ-Red recombinase system to replace the target gene and FLP recombinase for final excision of the antibiotic marker. The primers used to generate the knockout cassette were murIKOFW and murIKORV, including homology arms of 60 nt immediately upstream and downstream of the region to be deleted (Table 1). Hygromycin-sensitive single colonies that grew only on D-glutamate-containing plates were the mutant candidates. The *murI* deletion was confirmed by PCR with specific primers (EXTMURIFW and EXTMURIRV, Table 1) and sequence analysis.

The *murI* gene expression was analyzed by qRT-PCR in MGH 78578 and Δ*murI* strains with the primers listed in Table 1. The qRT-PCR protocol was performed as previously described [29]. The expression levels were normalized relative to the transcription levels of the *rpoB* housekeeping gene.

### 2.3. Growth and Viability Curves

Growth and viability of *K. pneumoniae* MGH 78578 and its *murI*-deficient mutant were determined as previously described [29]. Samples were taken at different times to measure the culture turbidity (optical density at 600 nm, OD_600_) and to determine the colony-forming units (CFUs) on LB agar supplemented or not with 10 mM D-glutamate. All cultures were prepared in triplicate.

### 2.4. Electronic Microscopy Analysis

Samples for scanning and transmission electron microscopy (SEM and TEM) were prepared in the absence or presence of 10 mM D-glutamate, as previously described [29]. Briefly, for SEM, the bacterial overnight cultures were washed with 0.9% NaCl, diluted 1:100 in LB and incubated at 37 °C with agitation for 2 h. Then, the cultures were washed again, diluted 1:100 in LB in the presence or absence of D-glutamate and incubated for another 2 h before fixation with 4% paraformaldehyde. After fixation, samples were washed and dehydrated in an ethanol series. For TEM, 2 or 3 colonies of each strain were plated onto LB agar supplemented or not with D-glutamate and incubated overnight at 37 °C. Then, the first streak of each plate was dissolved in PBS buffer, washed with cacodylate buffer and the cells were prefixed with 2.5% glutaraldehyde for 4 h and fixed with 1% osmium acetate. After dehydration with acetone, cells were embedded in SPURR, and ultrathin sections of these samples were obtained and examined.

### 2.5. Control of Phenotypic Stability

Overnight cultures of *K. pneumoniae* MGH 78578 Δ*murI* were diluted (1:100) in 100 mL of LB supplemented with 10 mM D-glutamate and incubated at 37 °C under agitation (180 rpm) for up to 8 days. Samples from the cultures were taken on different days, washed twice and plated on LB agar in the presence or absence of 10 mM D-glutamate for determination of cell viability. Agar plates were incubated at 37 °C for 4 days. Cultures were prepared in triplicate.

### 2.6. Water Survival Assay

The viability of *K. pneumoniae* strains in water was determined as previously described [29]. Samples of cultures were taken on different days until day 79 to determine the number of CFUs in LB agar (wild-type strain) and LB agar with 10 mM D-glutamate (Δ*murI* mutant strain). Cultures were prepared in triplicate.

### 2.7. In Silico Molecular Typing, Capsular Type and Resistance Profile Predictions

Multilocus Sequence Typing (MLST) was determined in silico using bacterial genomic data and the MLST 2.0 server available on www.genomicepidemiology.org (28 March 2022) and Kleborate software v2.2.0 (Melbourne, VIC, Australia) [30]. Antibiotic-resistance genes were screened using the Resfinder v4.1 software available from the Center for Genomic Epidemiology (https://cge.cbs.dtu.dk/services/ResFinder/ accessed on 28 March 2022) [31]. Capsular type (K-antigen) and O-typing were predicted in silico using Kaptive v2.0.0 (Melbourne, VIC, Australia) [32].

### 2.8. Ethics Statement

Animal experiments were performed according to the recommendations and the guidelines of the European Union (Directive 2010/63/EU) and current national legislation (RD 53/2013) on the protection of animals used for scientific purposes. Animals were bred and maintained under specific pathogen-free conditions in the facilities at the A Coruña University Hospital (CTF-XXIAC) and were provided with free access to food and water.

### 2.9. Mouse Experiments

Female BALB/c mice aged 6 to 9 weeks were used in this study. For inoculations, the *K. pneumoniae* MGH 78578 Δ*murI* strain was prepared as previously described [29], with minor modifications. In brief, the bacterial strain was grown in LB supplemented with 10 mM D-glutamate at 37 °C with shaking at 180 rpm until an OD_600_ of 0.7 was reached. Then, cells were harvested by centrifugation, washed and suspended in saline solution. The bacterial suspension was intraperitoneally (i.p.) injected (0.1 mL containing 4–6 × 10^6^ CFU unless otherwise indicated) into mice in a two-dose schedule (days 0 and 14). Serial dilutions of the inoculum were plated to verify actual CFUs delivered to the mice. For passive immunization tests, pools of sera were obtained from BALB/c mice (*n* = 5) i.p. administered three times with the vaccine candidate (2.6 × 10^7^ CFU) at 7-day intervals. Blood samples were taken from the submandibular vein or by puncture of the retro-orbital plexus as described in [29].

The protective efficacy of the vaccine was evaluated by i.p. challenge (0.1 mL) of control and immunized BALB/c mice (*n* = 6–8) with a lethal dose of virulent strains of *K. pneumoniae* on day 21. Clinical signs were examined twice-daily for a period of 7 days to measure disease severity and survival. Mice were euthanized 36 h post-infection, and the organs (spleens, livers and lungs) were aseptically removed, homogenized in sterile NaCl 0.9% and plated on LB agar for determination of CFU counts, in order to estimate bacterial dissemination to organs.

### 2.10. ELISA

The levels of total IgG and of subclasses IgG1, IgG2a, IgG2b and IgG3, and IgM antibodies were quantified in mouse serum using a whole-bacterial cell ELISA as previously described [29]. Cytokines IL-2, IL-4 and IL-17A, and IFN-γ were evaluated in the cellular supernatant of splenocytes isolated from immunized and control mice on day 55 after the second immunization. Mouse spleens were removed aseptically and mechanically disrupted. The cell suspension was enriched for lymphocytes by a gradient centrifugation. Then, splenocytes were ex vivo restimulated with the vaccine strain and incubated at 37 °C, 5% CO_2_ for 48 h. The cytokine levels were measured with a commercial ELISA kit (Affymetrix) according to the manufacturer’s instructions, with minor modifications [33].

### 2.11. Statistical Analysis

Mean values were compared using a Student’s *t*-test. Survival analysis was conducted using Kaplan–Meier curves and the Mantel–Cox log-rank test. Comparisons between pairs of groups were analyzed using the nonparametric and unpaired Mann–Whitney U test. *p*-values < 0.05 were considered statistically significant. Statistical analyses were performed using GraphPad Prism software package (version 6.01, GraphPad Prism Software Inc., San Diego CA, USA). Lethal doses were calculated using the probit analysis tool in the R package “ecotox” v1.4.4.

## 3. Results

### 3.1. Characterization of the K. pneumoniae MGH 78578 Glutamate Racemase-Deficient Mutant

**Figure 1:**
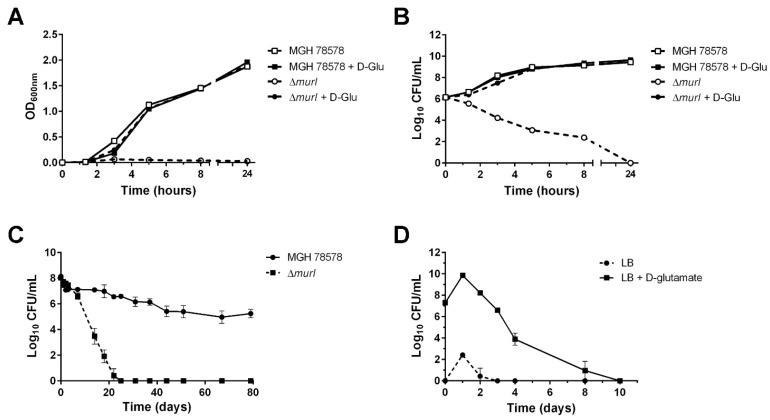
Growth (**A**) and viability (**B**) of *K. pneumoniae* MGH 78578 (squares) and the Δ*murI* mutant (circles) strain. Turbidity of cultures (OD at 600 nm) at different times and viability (Log_10_ CFU/mL) on LB agar containing 10 mM D-glutamate after growth with (solid symbols) or without (open symbols) D-glutamate. (**C**) Low persistence of D-glutamate auxotrophic strain in the environment. Viable counts (Log_10_ CFU/mL) of MGH 78578 (circles) and Δ*murI* mutant (squares) strains maintained in distilled water at 37 °C with agitation for 79 days. *p* = 0.0006, according to Student’s *t*-test. (**D**) Phenotypic stability of D-glutamate auxotroph MGH 78578. Viable counts recovered from LB agar (circles) or LB agar supplemented with 10 mM D-glutamate (squares) when this strain was grown on LB supplemented with 10 mM D-glutamate at 37 °C with shaking for 10 days. *p* = 0.0059, Student’s *t*-test.

Analysis of the genome sequence of the *K. pneumoniae* MGH 78578 strain revealed the existence of a single putative D-glutamate racemase gene: *murI* (KPN_04256). In order to produce D-glutamate auxotrophs, the *murI* gene was deleted from the chromosome of MGH 78578 via a λ-Red knockout system [28]. The *murI* deletion was confirmed by PCR with primers located upstream and downstream of the *murI* gene (Table 1): a 1189 bp fragment from the strain carrying the wild-type allele and a 470 bp fragment from the strain carrying the Δ*murI* allele (Figure S1). Moreover, the absence of *murI* gene mRNA in the mutant strain was confirmed by qRT-PCR (data not shown). MGH 78578 grew normally on LB agar without D-glutamate, while the MGH 78578 Δ*murI* mutant required D-glutamate supplementation for growth (Figure 1A). Moreover, a 5-log reduction in viable cell counts was observed after 5 h during incubation of the deletion mutant in LB without D-glutamate, and no viable bacteria were recovered from the culture after 24 h (Figure 1B).

The persistence of the D-glutamate auxotrophic strain in the environment was determined by survival analysis in water: the viability of the Δ*murI* mutant strain was considerably reduced, and no viable bacteria were recovered after 22 days in water. By contrast, the wild-type counterpart was still viable on day 79 (Figure 1C). There was a significant difference in water survival between the two strains (*p* = 0.0006, Student’s *t*-test).

To test the phenotypic stability of the nutritional auxotrophy of *K. pneumoniae* MGH 78578 Δ*murI*, cultures were grown in LB with 10 mM D-glutamate for 10 days and samples were taken on different days and plated on LB agar either with or without D-glutamate. Bacterial counts were significantly higher in supplemented plates at the initial incubation stage (day 0) and over the following days (Figure 1D) (*p* = 0.0059, Student’s *t*-test). The recovery of a few colonies in the medium without D-glutamate during the first days may be due to the use of accumulated D-glutamate in the cytoplasm during the initial growth in supplemented media. This difference shows that the Δ*murI* strain remains auxotrophic for D-glutamate over time.

**Figure 2:**
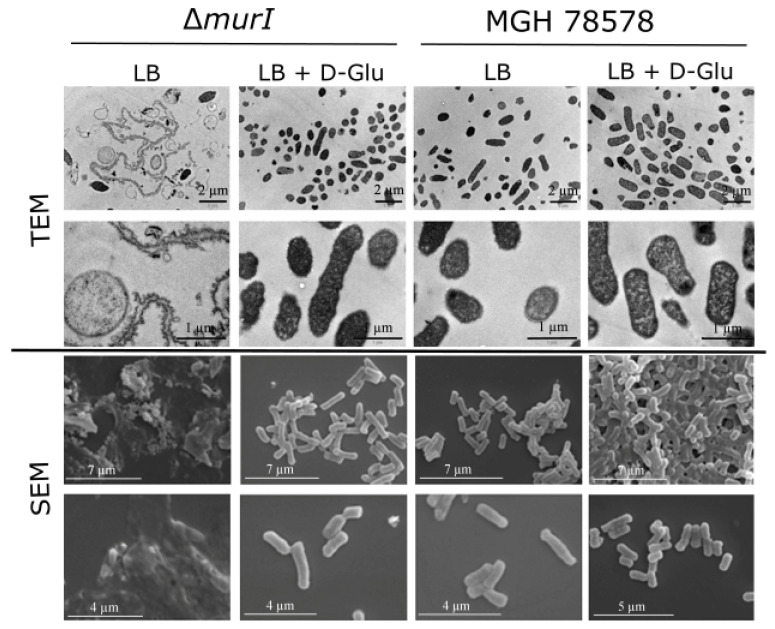
Morphological and structural changes of *K. pneumoniae* MGH 78578 Δ*murI* in the absence of D-glutamate. Transmission electron microscopy (TEM, upper panels) and scanning electron microscopy (SEM, lower panels) micrographs, obtained at different magnifications. Images of the wild-type strain are shown as controls.

Inspection of TEM and SEM micrographs showed that the Δ*murI* mutant is unable to divide in the absence of D-glutamate and has filamentous aggregates, protoplast-like structures and cellular debris (Figure 2). However, after D-glutamate supplementation at 10 mM, the Δ*murI* mutant cells had a similar appearance to their wild-type homologue in terms of both cell density and morphology.

### 3.2. The D-glutamate Auxotroph of K. pneumoniae Is Attenuated in BALB/c Mice

**Figure 3:**
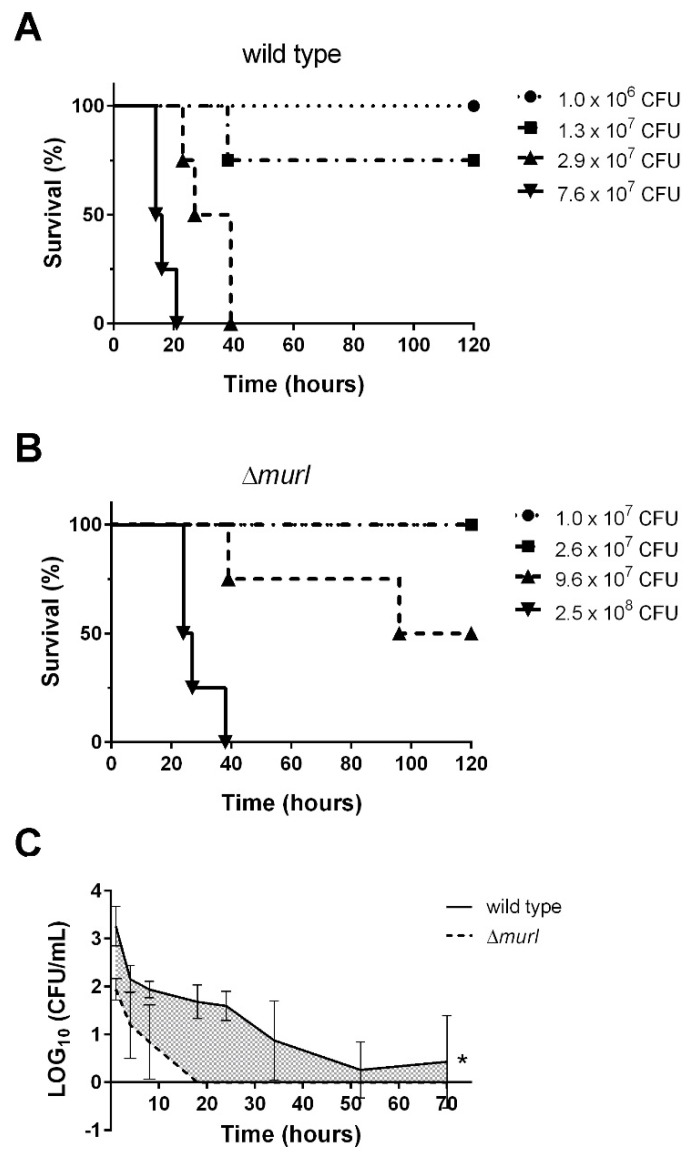
D-glutamate auxotrophic strain of *K. pneumoniae* MGH 78578 is attenuated for virulence in mice. Survival of BALB/c mice (*n* = 4) after i.p. injection with different doses of the MGH 78578 wild-type strain (**A**) and the Δ*murI* mutant strain (**B**). Mouse survival was monitored for 7 days. (**C**) Blood clearance of the D-glutamate auxotroph vaccine candidate after intravenous injection. Log_10_ CFU per mL of *K. pneumoniae* recovered from the blood of mice intravenously inoculated with 0.1 mL of the MGH 78578 wild-type (1.2 × 10^7^ CFU) and Δ*murI* strains (8.9 × 10^6^ CFU) over time. * *p* < 0.05, unpaired *t*-test.

Assessment of the impact on *K. pneumoniae* virulence revealed a marked decrease in the survival of mice inoculated with doses of 1.8 × 10^7^ CFU and higher of the wild-type strain. The lethal dose at which 50% of susceptible mice will die (LD_50_) of the wild-type strain MGH 78578 was calculated by the probit analysis to be 1.40 × 10^7^ CFU (Figure 3A). However, the estimated LD_50_ of the mutant strain Δ*murI* was 9.6 × 10^7^ CFU (Figure 3B).

In order to evaluate the safety of *K. pneumoniae* Δ*murI* as a vaccine, BALB/c mice either received an intravenous dose of the wild-type (1.2 × 10^7^ CFU; 0.1 mL) or Δ*murI* (8.9 × 10^6^ CFU; 0.1 mL) strains prepared in saline media. Blood samples were then collected from the mice at different times. The Δ*murI* strain was completely cleared from blood, with no colonies recovered beyond 18 h (Figure 3C), which suggests a high margin of safety for administration of the strain as a vaccine.

### 3.3. D-glutamate Auxotrophic Strain Generates Robust Humoral and Cellular Immune Responses against K. pneumoniae

Antibody-mediated immunity was evaluated after inoculation with the D-glutamate auxotroph to determine the minimum immunizing dose. As shown in Figure S2, significantly higher levels of antibodies against the wild-type strain were detected in all immunized mice compared with unvaccinated mice on days 7 and 14 (after one injection) and 21 (after two injections) (*p* < 0.005, Mann–Whitney U test). In contrast, on day 21 significant differences in IgG levels were observed between the group of mice inoculated with the dose of 7.2 × 10^4^ CFU and those in the control group (*p* < 0.005, Mann–Whitney U test), although to a lesser extent than with greater doses of vaccine. This demonstrates that a very low dose (100-fold lower than the dose of 7.9 × 10^6^ CFU) is adequate to trigger IgG production in mice, showing the immunogenic potential of the strain.

**Figure 4:**
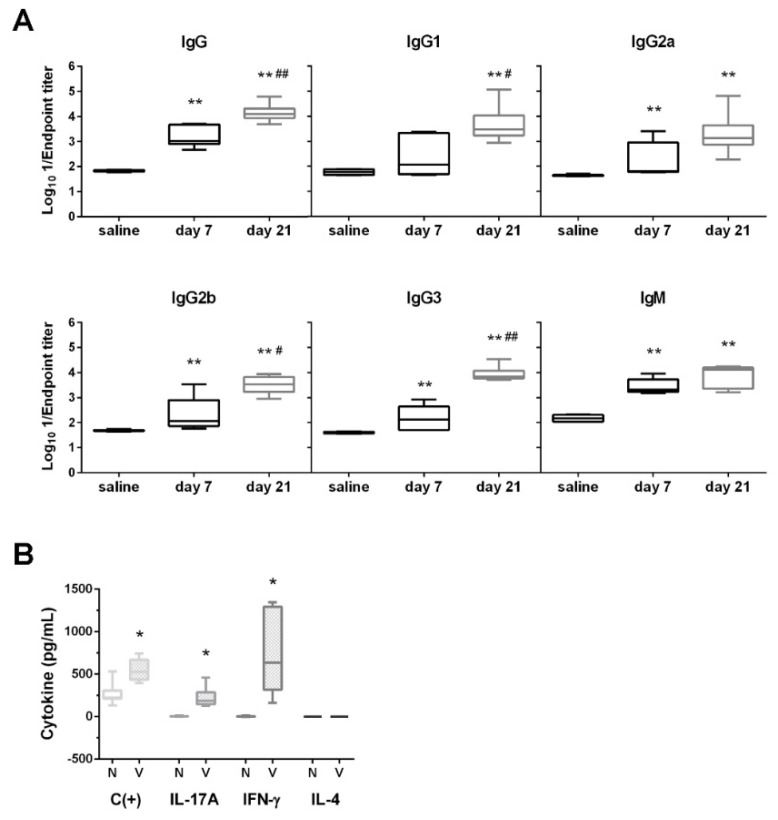
Humoral and cellular immune responses after inoculation. (**A**) Log_10_ 1/Endpoint titer of the IgG isotypes and IgM antibodies against the parental strain and produced in BALB/c mice (*n* = 6) on days 7 and 21 after one or two injections with MGH 78578 Δ*murI* (4.6 × 10^6^ CFU), and in non-vaccinated control mice (saline control). The antibody titers were determined by indirect ELISA. ** *p* < 0.005 relative to the uninoculated mice; # *p* < 0.05 and ## *p* < 0.005 relative to the preceding condition (Mann–Whitney U test). (**B**) Inoculation with the *K. pneumoniae* MGH 78578 D-glutamate auxotrophic strain triggered IFN-γ and IL-17A cytokine–secreting T-cells. BALB/c mice (*n* = 7) were immunized twice (days 0 and 14) with the auxotrophic strain (5 × 10^6^ CFU) or administered saline. On day 55 after the second inoculation, splenocytes were isolated and ex vivo restimulated (9 × 10^5^ cells per well) with the vaccine strain (4 × 10^6^ CFU per well) for 48 h. As a positive control (C+), mouse splenocytes were cultured with 1X Cell Stimulation Cocktail. Levels of IFN-γ and IL-17A in splenocyte supernatants differed considerably between vaccinated mice and naive mice (* *p* = 0.0001, Mann–Whitney U test).

In addition, on day 21 post-vaccination, significant levels of IgG and IgM immunoglobulins against the parent strain were present in all mice injected with the Δ*murI* strain (** *p* < 0.005, Mann–Whitney U test) and high levels of all IgG isotypes were determined (Figure 4A). Levels of all antibodies, except for IgG2a and IgM, were significantly higher after two immunizations (# *p* < 0.05 and ## *p* < 0.005, Mann–Whitney U test) than with one inoculation.

The ability of the aforementioned vaccine to stimulate cellular immune responses was assessed by measuring the secretion of cytokines in cellular supernatants of splenocytes. A strong production of IL-17A and IFNγ was observed after ex vivo antigen-specific restimulation with the vaccine strain, whereas no IL-4 was detected (Figure 4B).

### 3.4. The D-glutamate Auxotroph Vaccine Generates Cross-Protective Antibodies against K. pneumoniae Heterologous Strains

**Figure 5:**
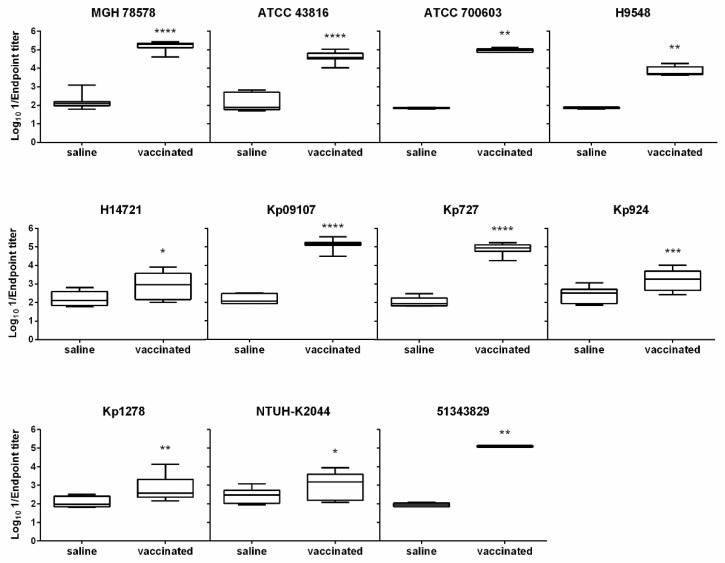
Inoculation with the D-glutamate auxotrophic vaccine elicited cross-reactive antibodies. Cross-reactivity (Log_10_ 1/Endpoint titer) of IgG antibodies produced by BALB/c mice (*n* = 6–12) on day 21 post-inoculation and in uninoculated control mice against the parental strain MGH 78578 and ten different *Klebsiella* spp. heterologous strains was observed. The antibody titers were determined by indirect ELISA. * *p* < 0.05, ** *p* < 0.01, *** *p* < 0.001 and **** *p* < 0.0001 relative to the group of uninoculated mice (Mann–Whitney U test).

The capacity of the vaccine strain to generate a broad immune response against the parental and several unrelated *Klebsiella* spp. strains, including MDR or hypervirulent clones belonging to different ST and K-types, was determined (Table 1, Tables S1 and S2). Significant IgG antibody titers were detected against all heterologous *K. pneumoniae* strains tested (*p* < 0.005, Mann–Whitney U test) (Figure 5). Highly significant IgG antibody titers were detected against five of these strains (ATCC 43816, ATCC 700603, Kp09107, Kp727 and 51343829), similar to that determined against the isogenic strain MGH 78578. These data demonstrate that inoculation with the Δ*murI* strain not only produces antibodies against the isogenic strain, but also induces IgG antibodies that cross-react with other unrelated *K. pneumoniae* and *K. quasipneumoniae* strains.

### 3.5. D-glutamate Auxotrophic Strain of K. pneumoniae MGH 78578 Elicits Protection against Infection with Different K. pneumoniae Strains

**Figure 6:**
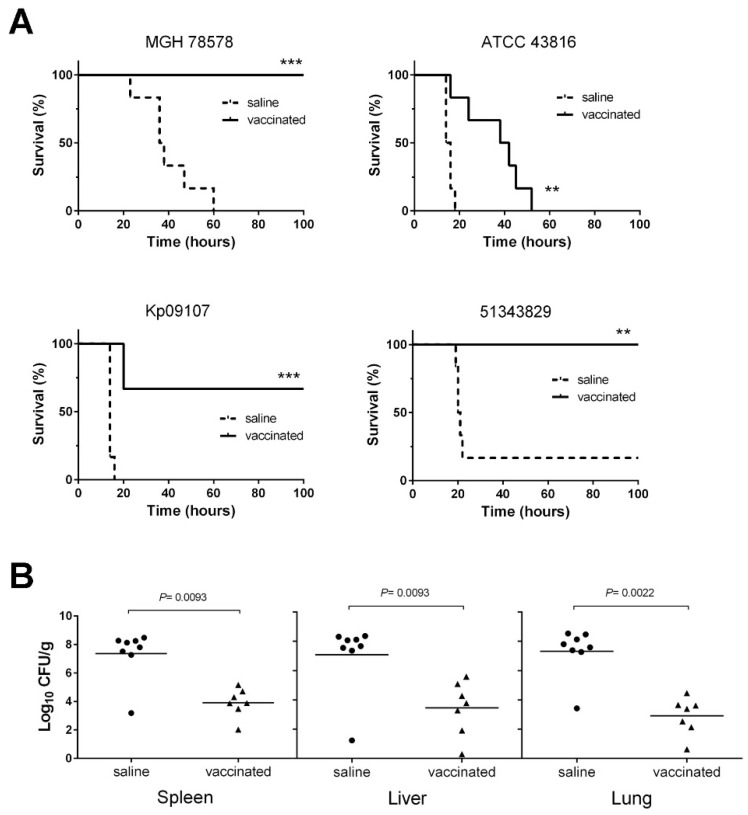
Vaccinated mice were protected from infection by *K. pneumoniae*. (**A**) Survival of BALB/c mice (*n* = 6) following i.p. infection with a lethal dose of different strains of *K. pneumoniae*: MGH 78578 (2.3 × 10^7^ CFU), Kp09107 (7.2 × 10^6^ CFU), ATCC 43816 (8.6 × 10^4^ CFU) and 51343829 (5.2 × 10^7^ CFU). Vaccinated mice were inoculated on days 0 and 14 with the MGH 78578 Δ*murI* strain (6.2 × 10^6^ CFU), while uninoculated mice were administered saline on the same days. Both mouse groups were infected with the wild-type strain on day 21. ** *p* < 0.005, *** *p* = 0.0005 survival of vaccinated group compared to the control group; *p*-values according to the Mantel–Cox log-rank test. (**B**) Bacterial loads in the spleens, livers and lungs obtained from immunized and control mice (*n* = 7–8) 36 h after infection with MGH 78578 (3.2 × 10^8^ CFU). Each symbol represents an individual mouse, and the horizontal lines show the averages for each group. *p*-values for the Mantel–Cox log-rank test.

To investigate whether vaccination with the Δ*murI* strain was sufficient to provide protection against *K. pneumoniae* lethal infections, BALB/c mice were challenged with other heterologous *K. pneumoniae* strains, including highly virulent strains and clinical isolates. When infected with 2.3 × 10^7^ CFU of the parental strain MGH 78578, all mice (*n* = 6) succumbed within the first 60 h after infection. By contrast, all vaccinated mice (*n* = 6) survived after overcoming the infection (*p* = 0.0005, Mantel–Cox log-rank test) (Figure 6A).

In the case of challenge with *K. pneumoniae* Kp09107, six deaths were reported in the group of unvaccinated mice during the first 16 h (*n* = 6; 100% mortality rate). By contrast, only two vaccinated mice died 20 h after infection (*n* = 6; 67% survival rate) Statistical analysis showed that survival differences between the two groups were highly significant (*p* = 0.0005, Mantel–Cox log-rank test). In the challenge with the ATCC 43816 hypervirulent strain, we found that all unvaccinated mice died within the first 18 h. Furthermore, the vaccinated mice inoculated with the ATCC 43816 strain survived for significantly longer (*p* < 0.005, Mantel–Cox test) and the death rate was slightly lower than in the unvaccinated mice. After challenge with the *K. pneumoniae* 51343829 strain (5.2 × 10^7^ CFU), five deaths were observed in the group of unvaccinated mice (83.3% mortality rate; *p* < 0.005, Mantel–Cox test). By contrast, all vaccinated mice survived this challenge and overcame infection (100% survival rate). For all of the strains tested, immunization with Δ*murI* significantly decreased or delayed mortality, relative to sham-immunization (log-rank Mantel–Cox test) (Figure 6A). Vaccination with this modified strain could therefore elicit protective immunity against infections caused by a diverse group of *K. pneumoniae* strains.

The passive transfer of anti-*K. pneumoniae* sera (anti-Kp) to naive mice by i.p. injection 3 h before challenge with MGH 78578 (2.6 × 10^8^ CFU) resulted in a significant level of survival in mice (62.5%; *p* < 0.05, log-rank test), while 87.5% of control mice that received naive serum succumbed to infection (Figure S3).

The bacterial load in different organs (spleens, livers and lungs) obtained from vaccinated mice after challenge with *K. pneumoniae* MGH 78578 (3.2 × 10^8^ CFU) was significantly lower than in the control group (at least a 3-log-unit reduction; *p* < 0.01, log-rank test), indicating that vaccination prevented bacterial dissemination to internal organs and the spread of systemic infection (Figure 6B).

## 4. Discussion

The increase in the occurrence of MDR, particularly ESBL-producing and carbapenem-resistant *K. pneumoniae*, is often associated with significant morbidity and high mortality rates among patients with bacteraemia [34,35]. In addition, the recent emergence of convergence of MDR and hypervirulent isolates and the lack of an effective vaccine against this pathogen [36,37,38] demands urgent efforts to accelerate research and development of new treatments and prevention strategies. The development of vaccines containing live attenuated strains auxotrophic for D-amino acids present in cell wall peptidoglycan is considered a promising approach to fight both Gram-positive and Gram-negative bacterial pathogens [29,33]. Like all whole-cell attenuated vaccines, our auxotrophic vaccines also exhibit complete coverage of bacterial epitopes, lower manufacturing costs and longer-term preservation; additionally, they are also safer, as replication is self-limited in the absence of specific requirements for growth. In this study, we constructed a glutamate racemase-deficient mutant of *K. pneumoniae* MGH 78578 for use as a potential vaccine against *Klebsiella* spp. infections. This Δ*murI* deletion mutant exhibited an absolute requirement of D-glutamate for growth and featured a stable auxotrophic phenotype. Interestingly, this vaccine candidate was shown to be less virulent than the parental wild-type strain and did not require an adjuvant to elicit a protective immune response. Importantly, this strain does not represent a risk for causing disease, as it is rapidly eliminated from the blood of mice in vivo.

We have shown that inoculation of mice with D-glutamate auxotrophic *K. pneumoniae* strain leads to high production of IgG and IgM antibodies. However, the IgG subclass distribution did not elicit predominance of the particular IgG isotype, suggesting a balanced Th1/Th2 profile. Higher levels of IgG3-specific antibodies were also induced in all the mice receiving the D-glutamate auxotrophic *K. pneumoniae* strain relative to the levels induced by DNA vaccines [39]. IgG1 and IgG3 responses are often linked during infection, and both antibodies can efficiently trigger the classical route of complement activation and promote opsonization [40,41]. Early IgG3 responses against protein antigens upon infection may be beneficial for the rapid clearance of pathogens [41]. The D-glutamate auxotrophic vaccine generates cross-reactive antibodies and induces a protective immune response against several heterologous *K. pneumoniae* strains, MDR or hypervirulent clones with different STs and K-types (Tables S1 and S2, Figure S4). However, lower serum IgG levels against isolates with CPS K1 and K24 were reached relative to the other strains tested. This result suggests that both the antibody production and a vaccine-induced T-cell response would be needed to fully protect against *K. pneumoniae* infections. In fact, we demonstrated an increase in IL-17A and IFN-γ production in response to immunization with the D-glutamate auxotroph vaccine. Th1 effector cytokines such as IFN-γ may play a crucial role in the resolution of *K. pneumoniae* lung infection by enhancing the antimicrobial activities of alveolar macrophages, resulting in bacterial clearance [42,43], while IL-17A may collaborate in promoting neutrophil recruitment and local control of pulmonary infection [44]. Vaccination with *K. pneumoniae*-derived extracellular vesicles was previously shown to elicit specific production of IFN-γ [45]. Likewise, a T-cell immune response mediated by IFN-γ, in addition to IL-4 and IL-17A, was also reported in mice vaccinated with some recombinant outer membrane proteins of *K. pneumoniae* [46].

In this study, the passive transfer of anti-Kp sera, elicited in response to inoculation with the D-glutamate auxotrophic strain, provided good levels of protection against systemic infection with the wild-type parental strain. In contrast, in the mid-1980s and the 1990s, other studies reported limited protection using anti-CPS antibody administered passively, e.g., optimal protection against fatal burn-wound sepsis was obtained using a combined antibiotic and passive anti-K1 CPS therapy regimen, and passive transfer of anti-K2 CPS reduced severity and inflammatory reactions in the lungs but did not prevent the invasion of virulent bacteria into the interalveolar space [47]. However, more recently, the protective effect against *K. pneumoniae*-induced lethality of adoptive serum and splenocyte transfers from mice vaccinated with extracellular vesicles was reported [45].

To our knowledge, this is the first time that a *K. pneumoniae* D-glutamate auxotroph has been tested as an experimental live vaccine against systemic *Klebsiella* spp. infections. Additional studies will be needed to demonstrate the ability of the D-glutamate auxotrophic mutant to stimulate protective immunity by using different routes of administration and *K. pneumoniae* infection models.

## 5. Conclusions

We have developed a vaccine prototype for the prevention of *Klebsiella*-induced sepsis. The vaccine consists of a live attenuated bacterial strain with auxotrophy for D-glutamate, a key structural component of bacterial cell walls. Our findings demonstrated that the candidate vaccine strain is safe and induces a protective immune response against systemic infections caused by *K. pneumoniae* strains, including MDR strains and some hypervirulent serotypes.

## Figures and Tables

**Table 1:** Bacterial strains, plasmids and primers used in this study.

Strain, Plasmid or Primer	Relevant Features or Sequence (5′ → 3′)	Reference
***K. pneumoniae*** **strains**		
MGH 78578	ATCC 700721, isolate from the sputum of a patient with pneumonia (ST38, K52)	American Type Culture Collection (ATCC)
MGH 78578 Δ*murI*	MGH 78578 derivative, ΔKPN_04256	This study
ATCC 43816	A K2 clinical pneumonia isolate (ST493, K2)	ATCC
ATCC 43816 KPPR1	A rifampin-resistant mutant of ATCC 43816	ATCC
ATCC 700603	*Klebsiella quasipneumoniae* (formerly known as *K. pneumoniae* K6), a clinical isolate from a hospitalized patient with urinary tract infection; ESBL reference strain	ATCC
H9548	A hypervirulent strain isolated from a patient with bacteraemia in Barcelona (ST493, K2)	[24]
H14721	A hypermucoviscous strain causing bacteraemia in adults in Barcelona (ST23, K1)	[24]
Kp09107	Isolate from rectal swabs of patients hospitalized in Spain (ST101, K17)	[25]
Kp727	Clinical isolate recovered from blood cultures in Spain (ST405)	[25]
Kp924	Clinical isolate from bronchoalveolar lavage fluid samples of patients in Spain (ST11, K24)	[25]
Kp1278	Isolate from multiple urine cultures in a hospital outbreak in Spain (ST15, K24)	[25]
NTUH-K2044	Isolate from a patient with liver abscess and meningitis in Taiwan (ST23, K1)	[26]
51343829	Hypermucoviscous strain from rectal swabs of patients in the A Coruña University Hospital (ST15)	Laboratory collection
***E. coli*** **strains**		
*E. coli*	DH5α competent cells (F^−^ φ80lacZΔM15 Δ(*lacZYA*-*argF*)U169 *recA1 endA1 hsdR17*(r_K_^−^, m_K_^+^) *phoA supE44* λ^−^*thi*-1 *gyrA96 relA1*)	Thermo Fisher Scientific
**Plasmids**		
pIJ773	pBluescript II SK(+) derivative containing an apramycin-resistance cassette (*aac(3)IV*) and the *oriT* from RK2 flanked by FLP recognition target (FRT) sites	[27]
pACBSR-Hyg	A p15A replicon plasmid containing an arabinose-inducible λ-Red recombinase and a hygromycin-resistance marker (*hph*)	[28]
pFLP-Hyg	A p15A replicon plasmid bearing a heat shock-inducible FLP recombinase and a hygromycin-resistance marker (*hph*)	[28]
**Primer**		
murIKOFW	CTGCAGGACGGGAATACACCTTGTCTGGCAGCTACACCTTCTGATCCACGTCCCACCATGATTCCGGGGATCCGTCGACC	
murIKORV	TGACAAGCCCTGTTTTCAAAAAATTATTCAACCGTCTCTTTTTTGTGCAAAACGCCCTTATGTAGGCTGGAGCTGCTTC	
EXTMURIFW	ATGATGATACTGGCAAGG	
EXTMURIRV	ATAGGGAGTTCTGAGACGT	
RT(KbrpoB) Fw	GGTGAAACTGCCTCCTTCG	
RT(KbrpoB) Rv	ACGGCCTTTCTCAACGTACA	
RT(KbMurI) Fw	GTGGGTTGTCGGTTTATAATGAG	
RT(KbMurI) Rv	GGCAACGTTATCGAAAGCATA	

## Data Availability

Data available upon request.

## Supplementary Materials

The following supporting information can be downloaded at: https://www.mdpi.com/article/10.3390/vaccines10060953/s1, Figure S1: PCR confirmation of the deletion in the Δ*murI* mutant of *K. pneumoniae* MGH 78578; Figure S2: Humoral immune response after inoculation; Figure S3: Passive anti-Kp sera transfer from immunized mice protects against *K. pneumoniae* infection in naive mice; Figure S4: Heatmap of in silico resistances detected using Resfinder; Table S1: Virulence, serotype, assembly metrics and ST predicted by Kleborate; Table S2: Chromosomic and acquired antimicrobial resistance (AMR) genes detected by Kleborate’s AMR module.
