# Supplementary material for: A New Live Auxotrophic Vaccine Induces Cross-Protection against Klebsiella pneumoniae Infections in Mice
Source: Vaccines (Basel). 2022 Jun 16;10(6):953. doi: 10.3390/vaccines10060953 (PMC9227041; doi:10.3390/vaccines10060953)
Supplement: Supplementary file 1 [file vaccines-10-00953-s001.zip › vaccines-1772512-supplementary.pdf]

A New Live Auxotrophic Vaccine induces Cross-Protection against *Klebsiella pneumoniae* Infections in Mice

Miriam Moscoso, Juan A. Vallejo, Maria P. Cabral, Patricia García, Víctor Fuentes-Valverde, Eva Gato, Jorge Arca-Suárez, Pablo Aja-Macaya and Germán Bou

Supporting Information

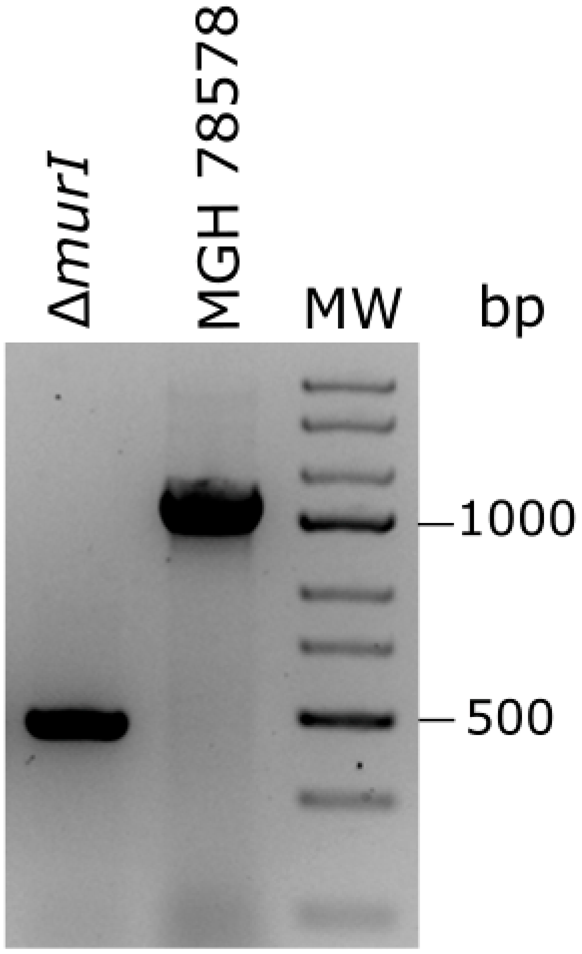

Figure S1. PCR confirmation of the deletion in the Δ*murI* mutant of *K. pneumoniae* MGH 78578. Oligonucleotides EXTMURIFW and EXTMURIRV were used to generate fragments with 1,189 bp from the strain carrying the wild-type locus KPN_04256 (*murI*) or a 470 bp fragment from the strains carrying the Δ*murI* mutant allele. MW, DNA molecular weight (in pb).

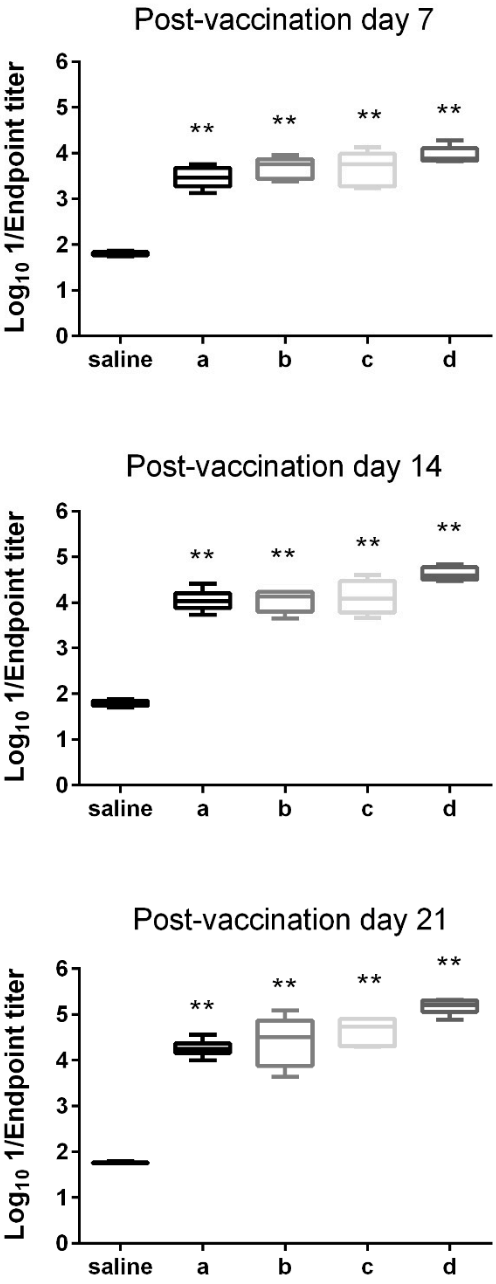

Figure S2. Humoral immune response after inoculation. Log_{10} 1/Endpoint titer of IgG antibodies produced against MGH 78578 in BALB/c mice (*n* = 6) on days 7, 14 (after one immunization) and 21 (two immunizations) post-inoculation with different doses of MGH 78578 Δ*murI* strain: 7.2×10^4 CFU (a), 3.5×10^5 CFU (b), 4.9×10^6 CFU (c) and 7.9×10^6 CFU (d), and in the uninoculated control mice (saline control). The antibody titers were determined by indirect ELISA. *$P < 0.05$ and **$P < 0.005$ (Mann-Whitney *U* test).

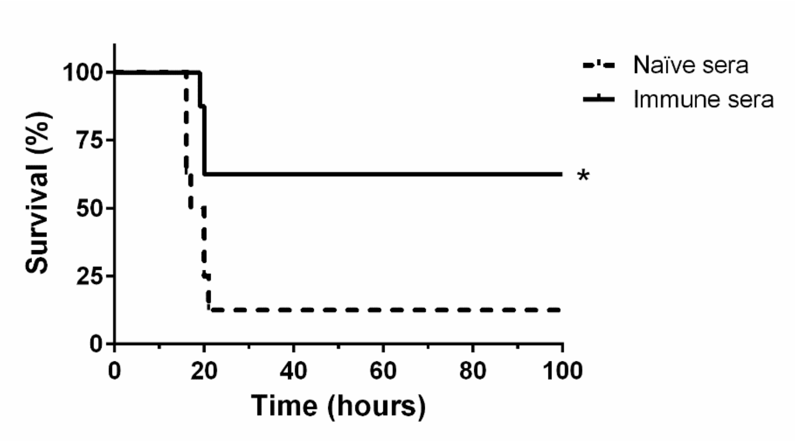

Figure S3. Passive anti-Kp sera transfer from immunized mice protects against *K. pneumoniae* infection in naive mice. BALB/c mice (n = 8) were administered with antisera (immune or naive serum) 3 hours before challenge with MGH 78578 (2.6×10^8 CFU) and survival was monitored daily for one week. **P* = 0.0298, log-rank test.

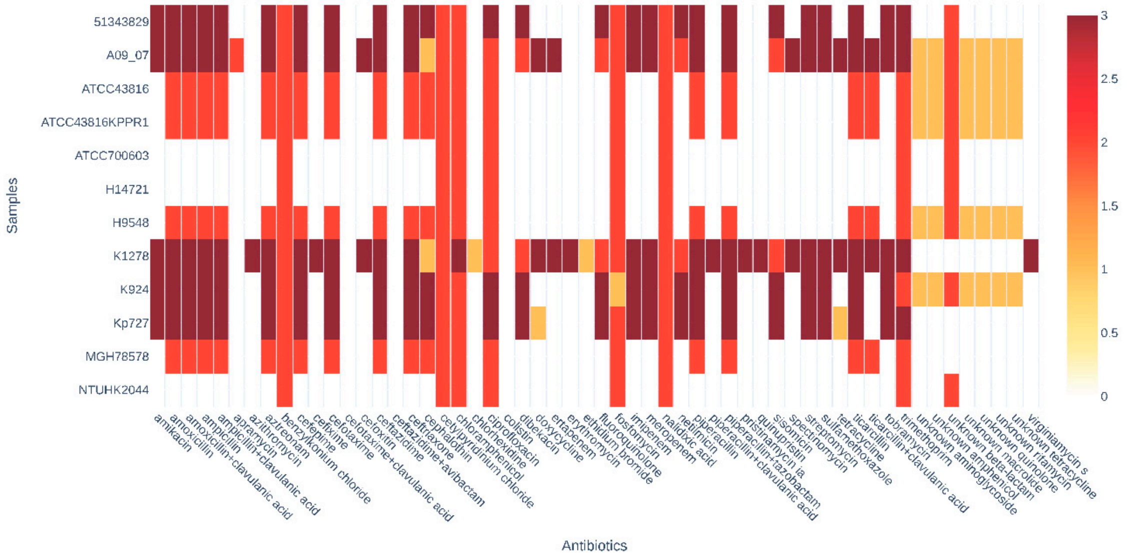

Figure S4. Heatmap of in-silico resistances detected using Resfinder. The level of resistance depends on if the genes detected are associated to AMR and on their similarity to the closest reference. White: No resistance detected; Yellow: partial match and length is smaller than reference; Bright red: full match but length is smaller than reference; Dark red: full match and equal length. The Resfinder v4.1 software is available in the Center for Genomic Epidemiology (https://cge.cbs.dtu.dk/services/ResFinder/).

Table S1. Virulence, serotype, assembly metrics and sequence type (ST) predicted by Kleborate software [1].

Strain	Species	ST	Contigs	N50	GenBank/BioSample Accession No.	Virulence score	wzi	K locus	K type	O locus	O type
**MGH 78578**	*Klebsiella pneumoniae*	ST38	1	5315120	CP000647.1	0	wzi50	KL52	K52	OL101	unknown (OL101)
**ATCC 43816**	*Klebsiella pneumoniae*	ST493	1	5362708	CP064352.1	1	wzi2	KL2	K2	O1/O2v1	O1
**ATCC 43816 KPPR1**	*Klebsiella pneumoniae*	ST493	1	5374834	CP009208.1	1	wzi2	KL2	K2	O1/O2v1	O1
**ATCC 700603**	*Klebsiella quasipneumoniae* subsp. *similipneumoniae*	ST489	1	5284734	CP014696.2	0	wzi171	KL53	K53	O3/O3a	O3/O3a
**H9548**	*Klebsiella pneumoniae*	ST493	46	224953	SAMN28747367	1	wzi2	KL2	K2	O1/O2v1	O1
**H14721**	*Klebsiella pneumoniae*	ST23	63	327040	SAMN28747366	5	wzi1	KL1	K1	O1/O2v2	O1
**Kp09107**	*Klebsiella pneumoniae*	ST101	1	5588067		1	wzi137	KL17	K17	O1/O2v1	O1
**Kp727**	*Klebsiella pneumoniae*	ST405	1	5700855	SAMEA1877770	1	wzi143	KL151	unknown (KL151)	O4	O4
**Kp924**	*Klebsiella pneumoniae*	ST11	1	5603904	SAMEA1877823	1	wzi24	KL24	K24	O1/O2v1	O2a
**Kp1278**	*Klebsiella pneumoniae*	ST15	174	198944	SAMEA1920272	0	wzi24	KL24	K24	O1/O2v1	O1
**NTUH-K2044**	*Klebsiella pneumoniae*	ST23	1	5248520	AP006725.1	1	wzi1	KL1	K1	O1/O2v2	O1
**51343829**	*Klebsiella pneumoniae*	ST15	105	184617	SAMN28747368	1	wzi93	KL112	unknown (KL112)	O1/O2v1	O1

1. Lam, M.M.C., et al., *A genomic surveillance framework and genotyping tool for Klebsiella pneumoniae and its related species complex.* Nat Commun, 2021. **12**(1): p. 4188.

Table S2. Chromosomic and acquired antimicrobial resistance (AMR) genes detected by Kleborate's AMR module.

Strain	MGH78578	ATCC43816	ATCC43816 KPPR1	ATCC700603	H9548	H14721	Kp09107	Kp727	Kp924	Kp1278	NTUH-K2044	51343829
**Aminoglycoside**	-	-	-	-	-	-	aac(3)-IId^; aac(6')-Ib'.v1; aadA*; strA.v1*; strB.v1*	aac(3)-IIa.v1^; aac(6')-Ib-cr.v2; strA.v1^; strB.v1	aac(6')-Ib-cr.v2; strA.v1^; strB.v1	aac(6')-Ib'.v1*; aadA*; strA.v1*; strB.v1*	-	aac(6')-Ib-cr.v2; strA.v1^; strB.v1
**Colistin**	-	-	-	-	-	-	-	-	-	-	-	-
**Fosfomycin**	-	-	-	-	-	-	-	-	-	-	-	-
**Fluoroquinolone**	-	-	-	-	-	-	-	qnrB1.v2^	qnrB1.v1	-	-	-
**Glycopeptide**	-	-	-	-	-	-	-	-	-	-	-	-
**MLS**	-	-	-	-	-	-	-	-	-	mphE.v2; msrE	-	-
**Phenicol**	-	-	-	-	-	-	catII.2*	CatB4.v1?	CatB4.v1; catII.2*	catA1^; catB2.v1	-	-
**Rifampin**	-	-	-	-	-	-	-	-	-	-	-	-
**Sulphonamide**	-	-	-	-	-	-	sul2	sul2	sul2	sul1; sul2	-	sul2
**Tetracycline**	-	-	-	-	-	-	tet(D)	-	-	tet(D)	-	-
**Tigecycline**	-	-	-	-	-	-	-	-	-	-	-	-
**Trimethoprim**	-	-	-	-	-	-	dfrA14.v2*	dfrA14.v2*	-	dfrA14.v2*; dfrB1	-	dfrA14.v2*
**Beta-lactamases**	-	-	-	-	-	-	OXA-9.v1	OXA-1; TEM-1D.v1^	OXA-1; TEM-1D.v1^	OXA-9.v1; SCO-1	-	OXA-1; TEM-1D.v1^
**Beta-lactamases with inhibitor resistance**	-	-	-	-	-	-	-	-	-	-	-	-
**ESBLs**	-	-	-	-	-	-	CTX-M-15	CTX-M-15	CTX-M-15	SHV-12	-	CTX-M-15
**ESBLs + inhibitor resistance**	-	-	-	-	-	-	-	-	-	-	-	-
**Carbapenemases**	-	-	-	-	-	-	KPC-2	OXA-48	OXA-48	VIM-1	-	OXA-48
**Chromosomic beta-lactamases**	SHV-11.v1^	SHV-1*	SHV-1*	OKP-B-6^	SHV-1	SHV-11.v1^	SHV-1^	SHV-76	SHV-11.v1	-	SHV-11.v1^	SHV-28.v1^
**SHV mutations**	35Q	-	-	-	-	35Q	-	35Q	35Q	238S; 240K; 35Q	35Q	-
**Omp mutations**	OmpK35-86%	-	-	-	-	-	OmpK35-61%; OmpK36-66%	OmpK35-70%	-	OmpK35-64%	-	OmpK35-4%; OmpK36-7%
**Col mutations**	-	-	-	-	-	-	-	-	-	MgrB-0%	-	-
**Flq mutations**	GyrA-83Y	-	-	-	-	-	GyrA-83Y; GyrA-87G; ParC-80I	-	GyrA-83F; GyrA-87A; ParC-80I	GyrA-83F; GyrA-87A; ParC-80I	-	GyrA-83F; GyrA-87A; ParC-80I

Mutations related to AMR are also represented. Each symbol represents the following: "^" means inexact nucleotide but exact amino acid match, "*" means inexact nucleotide and inexact amino acid match, "?" means incomplete match and "-X%" means truncated amino acid sequence.
